# Supplementary material for: Research Models to Study Ferroptosis’s Impact in Neurodegenerative Diseases
Source: Pharmaceutics. 2023 Apr 29;15(5):1369. doi: 10.3390/pharmaceutics15051369 (PMC10224233; doi:10.3390/pharmaceutics15051369)
Supplement: Supplementary file 1 [file pharmaceutics-15-01369-s001.zip › pharmaceutics-2303322-supplementary.pdf]

pharmaceutics

Supplementary Materials: Research models to study ferroptosis's impact in neurodegenerative diseases

Inês Costa, Daniel José Barbosa, Vera Silva, Sofia Benfeito, Fernanda Borges, Fernando Remião and Renata Silva

Table S1. Advantages and disadvantages of different *in vitro* models used in the study of neurodegenerative diseases.

Model	Advantages	Disadvantages
SH-SY5Y cells	Human origin. Capability of being differentiated into distinct neuronal phenotypes (cholinergic, dopaminergic or noradrenergic) [185]. Easy to maintain in culture. Suitable for high throughput screenings [94].	Genomic instability; metabolism and terminal differentiation influenced by its neuroblastoma origin [92]. Cellular outcome might depend on the differentiation protocol [94].
PC12 cells	Widely used for pharmacological studies. Easy to maintain in culture. Well established proliferation conditions and differentiation protocols [110]. Sensitive to toxins that disrupt mitochondrial function, including MPP^+, 6-OHDA and paraquat [92].	Rat origin. Derived from an adrenal pheochromocytoma, which may show distinct signalling pathways than those seen in neuronal cells [186].
LUHMES cells	Human origin. Exhibit dopaminergic features following differentiation [118].	Hard to transfect [92].
HEK293 cells	Human origin. High transfection efficiency. Easy to maintain in culture. Suitable for high throughput experiments [187].	Non-neuronal model. Genetic and epigenetic instability at higher passages [188].
NSC-34 cells	Present high degree of morphological and physiological properties of motor neurons. Easy to maintain in culture. Capability of being differentiated using different approaches. Suitable for high throughput screenings. May be sensitized to ferroptosis upon differentiation [128].	Mouse origin. Need differentiation to solve the lack of glutamate toxicity [189].
Organoids	More representative of the *in vivo* 3D brain environment. Show improved cellular maturity. High potential for CNS drug screening [92].	Low reproducibility. Require improved differentiation protocols and the establishment of an efficient vascularization. Ethical concerns [92].
iPSCs	Self-renewal potential. Potential to be differentiated into any cell type. Suitable for high throughput experiments Enable the *in vitro* mimicry of the disease microenvironment. Capability of being differentiated into dopaminergic neurons [190,191].	Difficult to establish the cellular outcome more representative of the disease pathogenesis. Require quality controls and the use of standardized protocols. Costly model [92].
Primary neuronal culture	Morphologically and physiologically comparable to human dopaminergic neurons	Cellular outcome might depend on the differentiation protocol.

		High potential for generating novel genetic models.	Low transfection efficiency and difficult to maintain in culture [92,186]. Ethical concerns.
Glial cell culture	Astrocytes	Suitable for repeated passages and amenable for cryopreservation.	Its growth is relatively slow, which makes it difficult to obtain [141].
	Microglia	Suitable for high throughput experiments.	Limited to the study of chronic diseases in animals and older humans.
	Oligodendrocytes	Can be differentiated and produce myelin in the absence of signals from axons. Easy to isolate and maintain.	Must be isolated from brain biopsies (in case of human oligodendrocytes), which leads to ethical problems [92].
Brain slices		Allows experimental manipulations that are complicate to perform *in vivo*. In the scope of MS can be demyelinated *in vitro* by exposure to toxins [92].	Several compounds that seem to be good candidates in this model, failed in human clinical trials.

6-OHDA: 6-hydroxydopamine; CNS: Central nervous system; iPSCs: Induced pluripotent stem cell lines; MPP^+: 1-Methyl-4-phenylpyridinium; MS: Multiple sclerosis.

Table S2. Advantages and disadvantages of *in vivo* models used in the study of neurodegenerative diseases.

Model	Advantages	Disadvantages
Rodents	Mimic the pathology. Possibility to create simulations of the pathology. Express orthologues of human proteins. Well established protocols.	Ethical concerns. Human and rodent brain with particular differences in what concerns to development and function [168].
Drosophila melanogaster	70% of genetic conservation for human disease-related genes [192]. Availability of numerous and well-established agents capable of inducing disease phenotypes (4-Vinylcyclohexene, Bisphenol A, among others) [193,194]. Easy access of compounds to the brain[192].	Highly divergent from human physiology [195–197].
Caenorhabditis elegans	Transparent body and precise neuronal anatomy [166]. Short lifespan (3 weeks) [166]. Fully sequenced and well-annotated genome, with ~38 % of the genes having human orthologues [198].	
Zebra fish	Display easily quantifiable behaviours. Simple, but well-conserved nervous system. Present a fully characterized genome, whose nucleotide sequence is comparable to other animal models [199,200]. 95% of genetic homology to humans, fast development and easy to breed [201]. Functional blood-brain barrier that is established 3 days after fertilization [202]. 84% of genetic conservation for human genes associated to dementia [203,204].	
Other animal models (dog, rabbit, pig)	Available for genetic modification to modeling ND. More similar with human organ physiology and anatomical dimensions [180].	Low efficiency of homologous recombination. Long-life cycle. Lack of embryonic stem cells for *in vitro* genome editing [181]. Ethical concerns [205]. Costly models.
